# Monitor alarms in the Emergency Department are frequent and unequally distributed during a day

**DOI:** 10.1186/1757-7241-23-S1-A41

**Published:** 2015-07-16

**Authors:** Thomas Schmidt, Camilla LN Bech, Marianne Glud, Uffe Kock Wiil, Annmarie Lassen

**Affiliations:** 1 The Mærsk Mc-Kinney Møller Institute, University of Southern Denmark, Odense M, Denmark; 2 Department of Emergency Medicine, OUH Odense University Hospital, Odense C, Denmark

## Background

Audible alarms from monitoring equipment in the Emergency Department add to the overall noise level in the department, may cause alarm fatigue of clinicians, and in the worst case to an undetected deterioration in a hospitalized patient. Our aim with this study is to describe the use of electronic monitoring in the Emergency Department, the distribution of generated audible alarms, and to uncover temporal patterns in monitoring.

## Methods

All adult patients admitted to the bed units of the Emergency Department at Odense University Hospital (November 15th 2013-August 1st 2014) with vital signs monitored as part of daily routine were included. Information from the monitors was stored in a research database. All registered vital signs were analyzed by hour of day in vital sign subgroups and compared with the generic alarm thresholds set in the Philips IntelliVue monitoring platform used at the department. We define an alarm event as three consecutive threshold violations.

## Results

A total of 3,825 patients were registered across 4,395 admissions. A total of 7,132,460 vital sign measurements were registered in patient contacts based on heart rate (19.2%), respiratory rate (19.3%), peripheral capillary oxygen saturation (30.3%), pulse rate (30.3%), systolic blood pressure (0.9%), and diastolic blood pressure (1.1%). The mean number of measurements/hour was 1,156 (SD = 211, 95% 1,067-1,246). The accumulated average number of alarm events triggered/hour was 19.1 (SD = 3.2, 95% CI 17.8-20.5). A total of 39.5% of the measurements were registered during the night, 31.9% during the day shift, and 28.6% in the evening shifts. Night-hours accounted for 37.9% of the alarm-events, 33.8% of alarm-events happened during the day and 28.3% of the total alarm-events happened in the evening hours.

## Conclusions

Although the monitor load increased during the night hours, the relative percentage of alarms generated went down. The alarm events generated by each vital sign type are distributed identically, but with different variation. Peripheral pulse rate and heart rate are the main contributors to daytime alarm events.

